# Synopsis of the Etiology and Treatment of Typhoid Fever

**Published:** 1865-09

**Authors:** Ross C. Russ

**Affiliations:** Royalton, Ind.


ARTICLE XXX.

### SYNOPSIS OF THE ETIOLOGY AND TREATMENT OF TYPHOID FEVER.

By ROSS C. RUSS, M.D., Royalton, Ind.

Authors in medical science differ very widely in reference to the origin of typhoid fever, so-called by Louis, or enteric, by Wood. Hence, different views in pathology and treatment. Wood's practice of medicine (vol. i, p. 322,) avers it to be an inherent predisposition or principle in the human system, and only needs some exciting cause to bring it into action, based upon the same hypothesis as some exciting cause will bring about the development of tuberculosis; whilst Watson believes it to be a blood-poison introduced into the system by a malarious influence, destroying the normal constituents and qualities of the blood, and rendering the blood unfit to nourish the different tissues of the body. But neither of these positions will do to base a successful practice upon; all treatment suggested by such pathology must undoubtedly end in empiricism.

What, then, is the true cause of typhoid fever? I believe it to be caused by poisonous gases in the blood, generated in the circulation—in other words, they are narcotic poisons that ought and must be eliminated from the economy by the excretory organs, in order that the various organs may perform their offices according to the design of nature. But I do not believe it to be malarious at all. If retained in the circulation, the blood becomes chemically deranged and impoverished. Hence, epistaxis, ecchymosis, in a word, all the vital functions that compose the organization become very much excited and their action depraved and perverted.

From a very careful study of typhoid fever, I am satisfied that the cause of derangement commences, doubtless, with the suspension of the functions of the skin and kidneys, which are the principal eliminating organs for the impurities of the circulation. Close the porosities of the skin to the escape of carbonic acid gas, and the kidneys to that of uric acid, and the result will be general poisoning of the whole organized structure of the body—the brain and nervous system receiving the greatest injury, because possessing the greatest degree of vitality.

That the nervous system manifests a great deal of derangement in this form of fever, we do not pretend to deny; but that tt is the seat and sole cause of disturbance, I certainly cannot believe. The nervous affection is certainly secondary to the primary impression, undoubtedly the effect and not the cause. It seems to me plainly manifested that it is an effort on the part of nature to eliminate from the circulation the impurities which it contains. Hence, a derangement of the nervous system, and also of the intestinal canal, &c.

Having arrived at these conclusions, as to the pathology and causes of typhoid fever, the true indications to be fulfilled in the treatment are to equalize the circulation, allay irritation, promote perspiration, and, in fact, aid the recuperative processes of nature. If we succeed in accomplishing this, we are able to arrest the fever at once, and restore the patient to a state of health. The various indications may be fulfilled by mild cathartics, diaphoretics, and diuretics. Standing preëminent in effecting these ends, mild cathartics will equalize the circulation, to a certain extent, and promote perspiration, and remove the unhealthy feculent accumulations of the bowels; anodyne diaphoretics allay irritation, and promote diaphoresis; diuretics aid in purifying the blood, by exciting the kidneys to a healthy action. When the skin is hot and dry, sponging with cold water will be found to afford great relief to the patient. If there is a liability to prostration and debility, tonics, stimulants, and a generous diet cannot be safely dispensed with, such as chicken or squirrel soup well salted, beef tea, &c.

